# Royal Delusions in Paranoid Schizophrenia: A Clinical Case with Chronic Evolution and International Repercussions

**DOI:** 10.1192/j.eurpsy.2026.12175

**Published:** 2026-07-31

**Authors:** P. Fernandez Perea, A. R. Diez, Y. Barrera Garcia, C. P. C. Alonso, R. Solorzano Vazquez, Á. F. Álvarez

**Affiliations:** Psychiatry , Hospital Leon, Leon, Spain

## Abstract

**Introduction:**

Paranoid Schizophrenia is a chronic disorder characterized by structured delusions, often of a persecutory or grandiose type, usually accompanied by poor insight and limited response to confrontation. Within this spectrum, royal or filiation delusions are rare but striking, as they provide a distinctive identity and can remain unchanged for decades. While such beliefs do not always affect daily functioning, in some cases they may drive unexpected behaviors with major consequences.

**Objectives:**

To describe the long-term evolution of a patient with Paranoid Schizophrenia and a persistent royal delusion, to analyze how apparently stable ideation may precipitate disruptive behavior, and to highlight the organizational implications for long-stay psychiatric care and international coordination.

**Methods:**

A descriptive, longitudinal single-case study was conducted using data from electronic health records, hospital documentation across different Spanish facilities, and direct observation during the current admission at Santa Isabel Psychiatric Hospital in León. Variables included sociodemographics, psychiatric history, delusional content, admissions, treatment, and clinical course.

**Results:**

The patient is a 56-year-old man, single, from Valladolid, diagnosed with paranoid schizophrenia for over 20 years. His first admission (2001–2012) occurred in a Penitentiary Psychiatric Hospital after violating a restraining order in the context of delusional jealousy. He later required multiple admissions in spanish hospitals of different cities (Valladolid, Madrid, León…) and periods in supervised housing. His psychopathology has consistently centered on a royal filiation delusion, claiming dynastic rights and multiple academic degrees. For long periods this belief had limited behavioral impact, but in January 2025, after three years of stability in a long-stay unit, he absconded intending to travel to Russia to present “new weapons projects” to President Putin. He reached Tallinn, Estonia, where he sought help at the Spanish Embassy, leading to admission in a local hospital and repatriation. On reassessment he was oriented, calm, and cooperative, with pressured but coherent speech containing grandiose and persecutory delusions. He denied suicidal or aggressive ideation. Current treatment is long-acting intramuscular olanzapine 405 mg/28 days, maintaining clinical stability under institutional supervision.

**Conclusions:**

This case illustrates the persistence of royal delusions in chronic paranoid schizophrenia, showing how beliefs apparently harmless for years can abruptly motivate high-impact behavior. It underlines the necessity of continuous supervision in long-stay facilities, the limitations of pharmacological treatment alone, and the importance of coordinated responses between mental health services and diplomatic institutions when delusion-driven actions transcend national boundaries.

**Disclosure of Interest:**

None Declared

